# Bone Mineral Density and Serum Levels of Bone Remodeling Markers in Ankylosing Spondylitis Treated with Anti TNF-α Agents

**DOI:** 10.3390/medsci13030189

**Published:** 2025-09-13

**Authors:** Efren Gerardo Alvarez-Ayala, Jorge Ivan Gamez-Nava, Ana Miriam Saldaña-Cruz, Fabiola Gonzalez-Ponce, Betsabe Contreras-Haro, Melissa Ramirez-Villafaña, Edsaul Emilio Perez-Guerrero, Miriam Fabiola Alcaraz-Lopez, Eli Efrain Gomez-Ramirez, Juan Manuel Ponce-Guarneros, Norma Alejandra Rodriguez-Jimenez, Sylvia Elena Totsuka-Sutto, Alberto Daniel Rocha-Muñoz, Luis Alfonso Muñoz-Miranda, Laura Gonzalez-Lopez, Cesar Arturo Nava-Valdivia

**Affiliations:** 1 Programa de Doctorado en Farmacología, Departamento de Fisiología, Centro Universitario de Ciencias de la Salud, Universidad de Guadalajara, Guadalajara 44340, Mexico; efren.alvarez2301@alumnos.udg.mx (E.G.A.-A.); ivangamezacademicoudg@gmail.com (J.I.G.-N.); 2 Instituto de Terapéutica Experimental y Clínica, Departamento de Fisiología, Centro Universitario de Ciencias de la Salud, Universidad de Guadalajara, Guadalajara 44340, Mexico; 3 Programa de Maestría en Salud Pública, Centro Universitario de Ciencias de la Salud, Universidad de Guadalajara, Guadalajara 44340, Mexico; 4 Departamento de Fisiología, Centro Universitario de Ciencias de la Salud, Universidad de Guadalajara, Guadalajara 44340, Mexico; 5 Unidad de Investigación Biomédica 02, UMAE, Hospital de Especialidades, Centro Médico Nacional de Occidente, IMSS, Guadalajara 44349, Mexico; 6 Instituto de Investigación en Ciencias Biomédicas, Centro Universitario de Ciencias de la Salud, Universidad de Guadalajara, Guadalajara 44340, Mexico; 7 Servicio de Reumatología, Hospital General Regional 46, Instituto Mexicano del Seguro Social, Guadalajara 44910, Mexico; 8 Departamento de Salud-Enfermedad Como Proceso Individual, Centro Universitario de Tonalá, Universidad de Guadalajara, Guadalajara 44100, Mexico; 9 Centro de Investigación en Enfermedades Infectocontagiosas, Departamento de Microbiología y Patología, Centro Universitario de Ciencias de la Salud, Universidad de Guadalajara, Guadalajara 44340, Mexico

**Keywords:** ankylosing spondylitis, bone mineral density, TNF-α, DKK-1, SOST, BMP-6, IL-17

## Abstract

**Background:** Ankylosing spondylitis (AS) is a chronic autoinflammatory rheumatic disease mainly affecting the sacroiliac joints and spine, causing altered bone remodeling. Pro-inflammatory cytokines such as TNF-α and IL-17 contribute to bone loss by modulating pathways including Wnt/β-catenin, which is inhibited by proteins like Dickkopf-1 (DKK-1) and sclerostin (SOST). Bone morphogenetic protein-6 (BMP-6) promotes osteoblast differentiation and bone formation. This study evaluated the association between serum levels of DKK-1, SOST, BMP-6, and bone mineral density (BMD) in AS patients treated with anti-TNF agents and conventional synthetic DMARDs (csDMARDs). **Methods:** A cross-sectional study included 76 AS patients diagnosed by modified New York criteria and 30 healthy donors matched by age and sex. BMD at the lumbar spine and hips was assessed by DXA in all participants. Disease activity (BASDAI) and functional index (BASFI) were measured in AS patients. Serum levels of DKK-1, SOST, BMP-6, TNF-α, and IL-17 were quantified by ELISA in both groups. AS patients were divided into two treatment groups: combined anti-TNFα and csDMARD therapy (*n* = 38), and only csDMARDs (*n* = 38). **Results:** Bone mineral density showed no significant statistical differences between the spine (*p* = 0.930) and hips (*p* = 0.876) in AS patients compared to healthy controls. The activity (BASDAI) and functionality (BASFI) scores were similar in both treatment groups (*p* = 0.161 and *p* = 0.271, respectively). No significant differences were found in serum levels of DKK-1 (*p* = 0.815), SOST (*p* = 0.771), BMP-6 (*p* = 0.451), or IL-17 (*p* = 0.335) between combined anti-TNFα and csDMARD therapy versus monotherapy with csDMARD. **Conclusions:** The combination of anti-TNF bDMARD therapy and csDMARD therapy is not significantly associated with serum levels of DKK-1, SOST, BMP-6, and BMD compared to those treated with csDMARD monotherapy in patients with AS. This study provides novel and clinically relevant evidence on how anti-TNF bDMARDs and csDMARDs differentially affect bone turnover biomarkers and bone health in patients with AS, contributing to a better understanding of therapeutic strategies and guiding future research and clinical decision-making.

## 1. Introduction

Ankylosing Spondylitis (AS) is an autoinflammatory rheumatic disease that mainly affects sacroiliac joints and the lumbar spine [1,2]. Patients in the course of AS require a basic pharmacologic scheme of conventional synthetic disease-modifying antirheumatic drugs (csDMARDs), such as methotrexate, sulfasalazine, and azathioprine, inhibiting different pathways of the immune system to reduce inflammation and autoimmune activity. Moreover, biologic disease-modifying antirheumatic drugs (bDMARDs) have been included in the treatment when AS activity does not decrease. Pharmaceuticals with the highest prescription include Etanercept, Adalimumab, Infliximab, and Golimumab, whose mechanism of action is based on blocking the receptor–ligand interaction of tumor necrosis factor-alpha (TNF-α), a key inflammatory protein in AS [3]. TNF-α is the principal cytokine involved in autoinflammation in AS; it can synergize with Interleukin-17 (IL-17) expression, activating osteoclast precursors and diminishing bone mineral density (BMD) [4].

Low BMD has been reported in 20% of lumbar spine and 30% of total hips in Mexican AS patients [5]. Recently it has been shown that different molecules associated with the chronic inflammation process alter the intracellular Wnt/β-catenin signaling pathway, modifying the bone remodeling cycle. In addition, AS patients, beyond presenting low BMD, show a high risk of vertebral fractures that impair spinal function, cause severe pain, and reduce mobility, significantly compromising quality of life and underscoring the need to monitor bone fragility in this population [2,5].

A close relation has been demonstrated between chronic inflammation produced by TNF-α and signaling pathways such as the Wnt signaling pathway and different molecules that antagonize it, as well as the result in the production of bone turnover molecules [6]. In turn, it can also be affected by Dickoppf-1 (DKK-1) and Sclerostin (SOST), proteins produced by osteocytes, capable of interacting with membrane co-receptors LRP5/6 in osteoblast precursor cells, avoiding dimerization with the receptor coupled to G protein; consequently, the signaling pathway is interrupted, waning the osteoblast differentiation and decreasing bone formation [7,8].

Given their inhibitory effect on the Wnt/β-catenin pathway, modulation of DKK-1 and SOST could theoretically shift the balance of bone remodeling and counteract the excessive bone loss observed in AS. In contrast, proteins of the Bone Morphogenetic Protein (BMP) family play an important regulatory role in bone turnover and formation in AS. Among them, BMP-6 is of particular interest. Although BMPs are widely recognized for their role in osteoblast differentiation and bone metabolism, BMP-6 has been less extensively studied in rheumatic diseases. Its expression in osteocytes and osteoclasts suggests a potential contribution to bone remodeling, and its capacity to stimulate osteoblast differentiation may theoretically counterbalance the bone loss associated with AS [9].

While anti-TNF therapy has proven efficacy in reducing inflammation and improving function in AS, its impact on bone metabolism and BMD remains unclear, with conflicting evidence from previous studies. The objective of this study was to investigate whether treatment with anti-TNF agents, alone or in combination with csDMARDs, influences serum levels of DKK-1, SOST, BMP-6, and BMD in patients with AS.

## 2. Materials and Methods

### 2.1. Study Design

Cross-sectional study.

### 2.2. Study Subjects

Seventy-six AS patients were ≥18 years of age, diagnosed according to the modified New York criteria for AS in 1984, with a minimum disease duration of one year, and were derived from the Institute of Experimental and Clinical Therapeutics (INTEC, University of Guadalajara). We excluded patients with overlapping syndrome, hyperthyroidism, hyperparathyroidism, as well as infections such as hepatitis B/C, HIV, or tuberculosis, and any oncologic process. A total of thirty healthy donors from the same institute were enrolled as the age- and sex-matched control group. All controls underwent prior screening to rule out osteoporosis, metabolic bone disorders, chronic diseases, or any history of pharmacological treatments affecting bone metabolism. Concomitant use of glucocorticoids was permitted; however, calcium and vitamin D supplementation were not allowed. Body Mass Index (BMI) was not considered an exclusion criterion. All participants meeting the inclusion criteria were enrolled after providing written informed consent. All study procedures were carried out in accordance with the Declaration of Helsinki (Fortaleza, Brazil, 2013) and were approved by the Research and Ethics Committee of the University of Guadalajara (Code of approval: CI-08520, 2 November 2020).

### 2.3. Clinical Setting

We examined the epidemiological and clinical characteristics as well as pharmacological treatments (bDMARDs and csDMARDs) in all participants. We assessed AS patients’ disease activity using the Bath Ankylosing Spondylitis Disease Activity Index (BASDAI) [10] and functional disability using the Bath Ankylosing Spondylitis Functional Index (BASFI) [11]. Groups were divided by treatment as follows: (1) thirty-eight AS patients under bDMARDs (anti-TNFα) and csDMARDs, and (2) thirty-eight AS patients under only csDMARDs.

### 2.4. Bone Mineral Density (BMD) Measurements

BMD was evaluated by dual-energy X-ray absorptiometry (DXA) using a LUNAR Prodigy 2000 densitometer (GE Medical Systems Lunar, Madison, WI, USA) following standardized protocols. We obtained the total hip and lumbar spine (L1–L4) BMD results (gr/cm^2^) in all subjects and classified them employing World Health Organization criteria [12,13]. All DXA measurements were performed by a certified operator, and the densitometer was routinely calibrated and maintained by the manufacturer to ensure accuracy and reproducibility of the results. 

### 2.5. Quantification of Serum DKK-1, SOST, BMP-6, and Cytokines Levels

Trained personnel collected blood samples early in the morning following an 8-hour fasting period. The samples were obtained through venipuncture and collected in sterile, dry collection tubes. Serum was obtained through centrifugation at 3500 rpm and then preserved at −80 °C until it was processed.

Serum DKK-1, SOST, and BMP-6 concentrations were quantified by enzyme-linked immunosorbent assay (ELISA) using commercial kits: DKK-1 (Human Dkk-1 Quantikine ELISA Kit, R&D Systems, Minneapolis, MN, USA; sensitive assay of 0.948 pg/mL, range of 9.4 to 600 pg/mL), SOST (Human SOST/Sclerostin Quantikine ELISA Kit, R&D Systems, Minneapolis, MN, USA; sensitive assay of 3.8 pg/mL, range of 31.3 to 2000 pg/mL), and BMP-6 (Human Bone Morphogenetic Protein 6 ELISA Kit, MyBioSource, San Diego, CA, USA; sensitivity assay of 9.38 pg/mL, range of 15.6 to 1000 pg/mL).

Additionally, serum cytokines TNF-α and IL-17 were measured by the same analytic method. TNF-α (Human TNF-alpha Quantikine ELISA Kit, R&D Systems, Minneapolis, MN, USA; sensitivity assay of 6.23 pg/mL, range of 15.6 to 1000 pg/mL) and IL-17 (Human IL-17 Quantikine ELISA Kit, R&D Systems, Minneapolis, MN, USA; sensitivity assay of 15 pg/mL, range of 31.2 to 2000 pg/mL).

Serum concentrations of all molecules were determined by spectrophotometry reading at 450 nm using an ELISA microplate reader (Multiskan FC Thermo Fisher Scientific Inc., Tijuana, B.C., Mexico). All samples were assessed in triplicate according to the manufacturer’s guidelines.

### 2.6. Statistical Analysis

Non-parametric statistics were employed based on data distribution. Quantitative variables were expressed as medians and min-max, while qualitative ones were expressed as frequencies and percentages (%). The comparison between qualitative and quantitative variables of AS patient groups was evaluated using the chi-square test and the Mann–Whitney U test, respectively. The Kruskal–Wallis test and Bonferroni post hoc test were performed for comparison between the AS patient group and the control group. For correlation analysis between bone turnover biomarkers and BMD, the Spearman correlation test was employed. The level of significance was defined as *p* ≤ 0.05. The statistical analysis was conducted using the Statistical Package for the Social Sciences (SPSS) software for Windows, version 27.0 (SPSS Inc., IL, USA).

## 3. Results

### 3.1. Characteristics of AS Patients

We examined 76 patients diagnosed with AS, separated in two groups: 38 using csDMARDs + anti-TNF bDMARDs and 38 with csDMARDs. Median age was 49 (18–69) years and 10 (1–33) years of disease evolution in AS patients. The clinical BASDAI index showed a median score of 5.35 (0.4–9.5), indicating that patients had active disease; therefore, the BASFI median score was 3.9 (0–10.0), which proved loss of functionality at the time of the study. Moreover, 24 patients (31.6%) registered low BMD in the lumbar spine and 16 (21.1%) in total hip, Table 1.

All AS patients were treated with synthetic DMARDs (*n* = 76), of which 71.1% received sulfasalazine, 31.6% methotrexate, and 21.1% azathioprine. Also, 22.4% received combination therapy with 2 or more conventional synthetic DMARDs. In addition, 32.9% received glucocorticoids and 78.9% non-steroidal anti-inflammatory drugs (NSAIDs). Of the group of patients with anti-TNF treatment (*n* = 38), 40% received etanercept, 5.3% adalimumab, 7.9% infliximab, and only 2.6% received golimumab. At most 3.9% of patients with anti-TNF biological treatment received 2 or more biologicals simultaneously. 

### 3.2. Comparison of Characteristics Between AS vs. Healthy Donors

In the comparison of characteristics between AS vs. healthy donors, significant differences were observed in BMI [26.5 kg/m^2^ (16.40–40.86) vs. 28.75 kg/m^2^ (20.19–40.96), *p* = 0.014], lumbar spine low BMD prevalence [31.6% vs. 6.7%, *p* = 0.024], total hip BMD [0.94 g/cm^2^ (0.634–1.30) vs. 1.06 g/cm^2^ (0.81–1.34), *p* < 0.001], serum BMP-6 levels [2.05 pg/mL (0.41–52.77) vs. 21.44 pg/mL (12.17–50.0), *p* = 0.001], and TNF-α concentrations [8.87 pg/mL (0.01–938.19) vs. 4.66 pg/mL (0.01–18.4), *p* < 0.001]. No statistically significant differences were found for sex distribution, age, BMD in the lumbar spine, prevalence of low BMD for total hips, DKK-1, SOST, or IL-17 levels, Table 2.

### 3.3. Comparison of Characteristics, Serum DKK-1, SOST, BMP-6, and Cytokines in AS Study Groups

Table 3 shows the comparative analysis between group 1 (bDMARDs anti-TNF + csDMARDs) and group 2 (csDMARDs). Male gender was more prevalent in the group with bDMARDs + csDMARDs [27 (71.1%) vs. 19 (50%), *p* = 0.050]; patients in this group were younger [43 years (18–69) vs. 53 years (24–66), *p* = 0.007] but similar in years of disease evolution [10 years (1–33) vs. 10 years (1–30), *p* = 0.625]. Activity and functionality indexes do not show statistical differences between both groups [5.1 score (0.4–9.5) vs. 5.9 score (1.4–9.2), *p* = 0.285, and 3.7 score (0–9.5) vs. 3.9 score (0.8–10.0), *p* = 0.394]. Therefore, there were no differences in BMD for lumbar spine 1.11 gr/cm^2^ [0.75–1.68) vs. 1.10 (0.79–1.55), *p* = 0.930] and total hips 0.94 gr/cm^2^ [0.66–1.30) vs. 0.93 (0.63–1.26), *p* = 0.876]. There were no differences in low BMD and serum levels for DKK-1 [868.45 pg/mL (13.77–6748.21) vs. 730.85 (31.30–8434.54), *p* = 0.815], SOST [63.09 pg/mL (7.19–299.12) vs. 65.82 (6.48–357.32), *p* = 0.771], BMP-6 [3.34 pg/mL (0.41–52.77) vs. 1.60 (0.58–41.16), *p* = 0.451], and IL-17 [5.35 pg/mL (2.84–69.07) vs. 5.58 (1.65–62.39), *p* = 0.335]. In patients with anti-TNF and csDMARD therapy vs. patients only with csDMARD therapy, only serum TNF-α levels showed statistically significant differences [17.42 pg/mL (0.013–350.60) vs. 7.02 (0.72–938.19), *p* = 0.017*] [Table 3].

Moreover, in the comparison between AS study groups and healthy donors, we observed statistically significant differences in serum BMP-6 levels [3.34 pg/mL (0.41–52.77) vs. 1.60 pg/mL (0.58–41.16) vs. 21.44 pg/mL (12.17–50.0, *p* = 0.005], TNF-α [17.42 pg/mL (0.013–350.60) vs. 7.02 pg/mL (0.72–938.19) vs. 4.66 pg/mL (0.013–18.40), *p* < 0.001] and BMD in total hips [0.95 gr/cm^2^ (0.66–1.30) vs. 0.93 gr/cm^2^ (0.63–1.26) vs. 1.06 gr/cm^2^ (0.81–1.34), *p* = 0.003]. But not in DKK-1 [868.45 pg/mL (13.77–6748.21) vs. 730.85 pg/mL (31.30–8434.54) vs. 862.60 pg/mL (368.0–1710.0), *p* = 0.719], SOST [63.09 pg/mL (7.19–299.12) vs. 65.82 pg/mL (6.48–357.32) vs. 67.85 pg/mL (17.5–167.80), *p* = 0.888], IL-17 [5.35 pg/mL (2.84–69.07) vs. 5.58 pg/mL (1.65–62.39) vs. 4.92 pg/mL (2.4–28.2), *p* = 0.429], nor BMD in Lumbar Spine [1.11 gr/cm^2^ (0.75–1.68) vs. 1.10 gr/cm^2^ (0.79–1.55) vs. 1.18 gr/cm^2^ (0.92–1.44), *p* = 0.226]. 

### 3.4. Post Hoc Analysis

**Figure 1:**
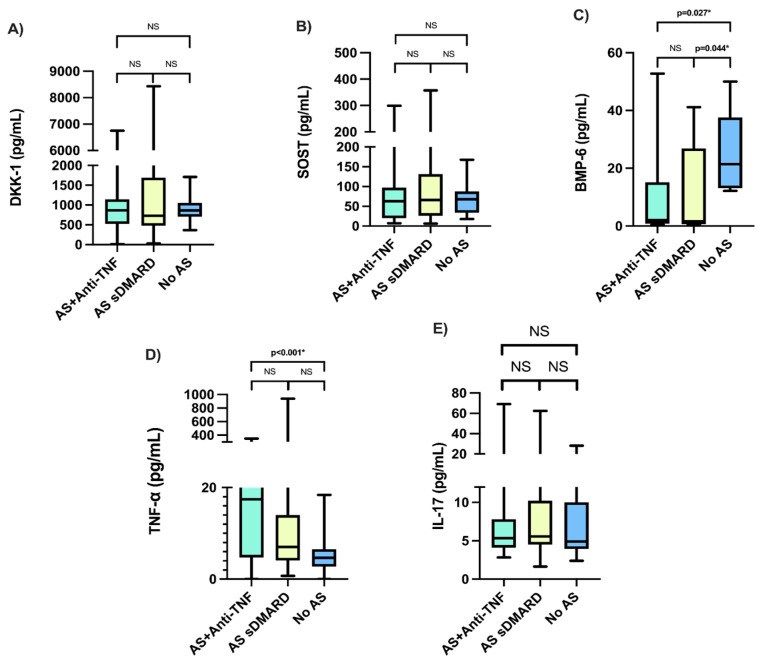
Multiple comparisons of the quantitative serum levels of the proteins of interest be-tween group 1 (bDMARDs anti-TNF + csDMARDs), group 2 (csDMARDs), and group 3 (control). (**A**) Comparison of serum DKK-1 levels between groups; (**B**) Comparison of serum SOST levels; (**C**) Comparison of serum BMP-6 levels; (**D**) Comparison of serum TNF-α levels; (**E**) Comparison of serum IL-17 levels. NS No statistical significance. * Statistical significance *p* ≤ 0.05.

A Kruskal–Wallis test was made to identify differences in DKK-1, SOST, TNF-α, BMP-6, and IL-17 between study groups. There were no statistical differences for DKK-1 (*p* = 0.719), SOST (*p* = 0.888), and IL-17 (*p* = 0.429), but BMP-6 (*p* = 0.005) and TNF-α (*p* ≤ 0.001) did show significant differences. Following these results, a Games–Howell post hoc test was implemented, showing differences for BMP-6 between patients with AS using bDMARDs + csDMARDs vs. those using only csDMARDs (*p* = 0.044) and no AS (*p* = 0.027), and for TNF-α in patients with AS using bDMARDs + csDMARDs vs. the csDMARDs only group vs. no AS (*p* ≤ 0.001), Figure 1.

### 3.5. Correlation Analysis

In our population, we observed a positive correlation of serum levels of DKK-1 with lumbar spine BMD (Rho = 0.369, *p* = 0.022), but not with total hips. No correlation was observed between SOST levels for any anatomical region. BMP-6 levels were positively correlated with total hip BMD (Rho = 0.736, *p* = 0.012). Regarding serum levels of cytokines, TNF-α showed no correlation with BMD, while IL-17 showed a positive correlation with lumbar spine BMD (Rho = 0.438, *p* = 0.108) and BMD in total hips (Rho = 0.486, *p* = 0.004).

**Figure 2:**
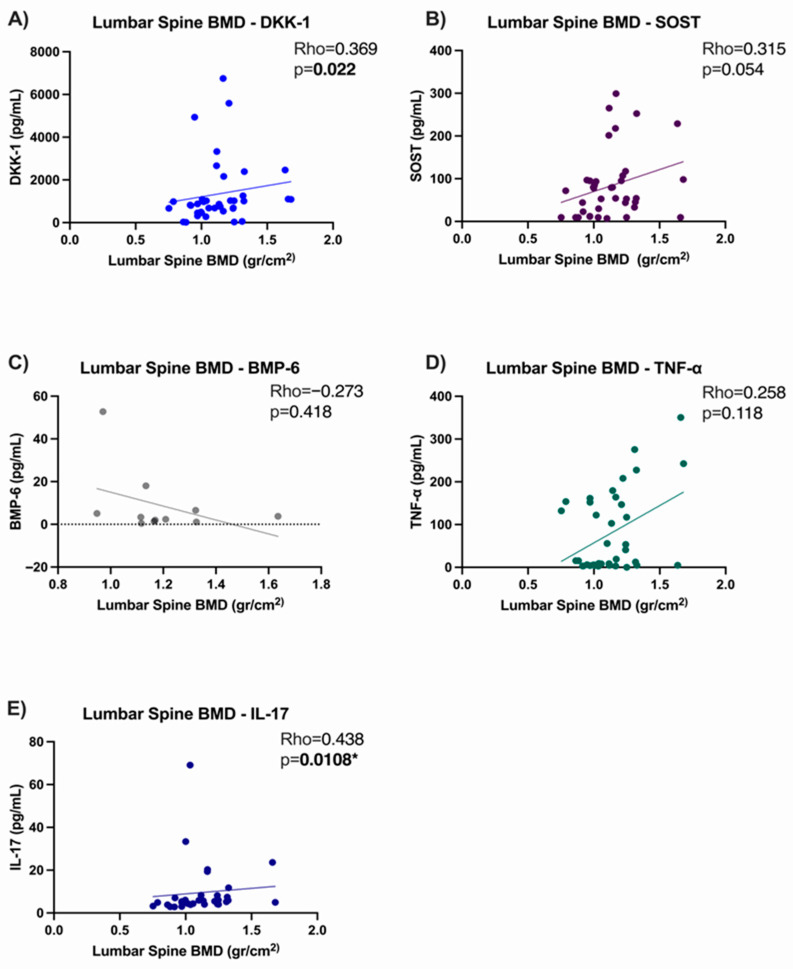
Correlation analysis between mineral density in lumbar spine and serum levels of the molecules evaluated in AS patients treated with anti-TNF. (**A**) Correlation between lumbar spine BMD and DKK-1; (**B**) Correlation between lumbar spine BMD and SOST; (**C**) Correlation between lumbar spine BMD and BMP-6; (**D**) Correlation between lumbar spine BMD and TNFα; (**E**) Correlation between lumbar spine BMD and IL-17. * Statistical significance *p* ≤ 0.05.

Serum DKK-1 levels in group 1 (bDMARDs anti-TNF + csDMARDs) showed a positive correlation with SOST levels (Rho = 0.654, *p* ≤ 0.0001). While serum BMP-6 showed a negative correlation with serum SOST (Rho = −0.836, *p* = 0.003). Moreover, there was a significant relationship between BMP-6 and TNF-α (Rho = 0.688, *p* = 0.024), Figure 2.

**Figure 3:**
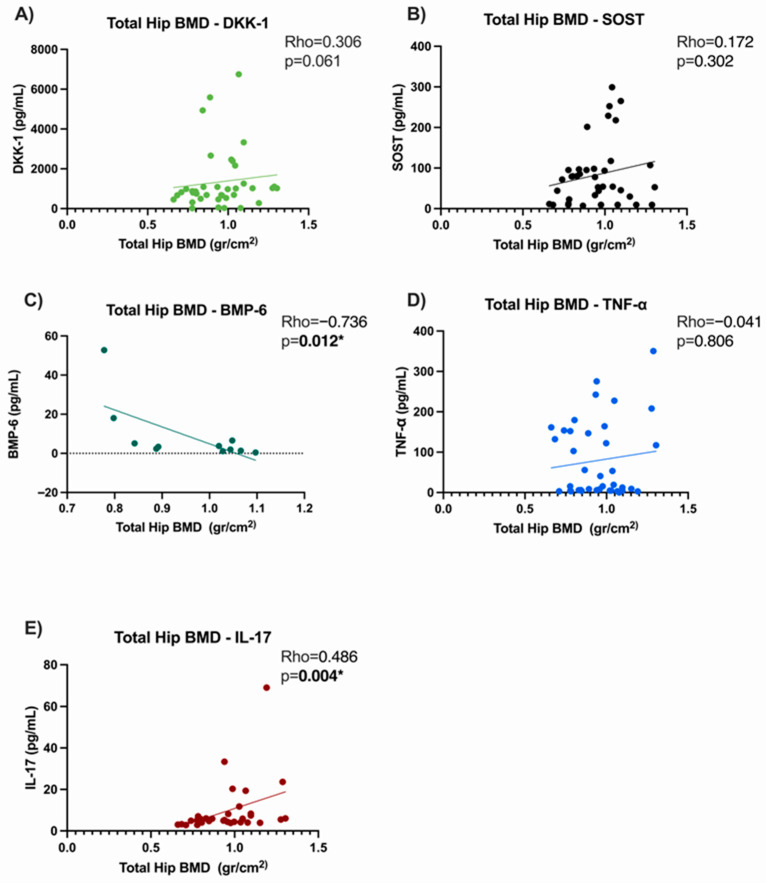
Correlation analysis between mineral density in total hip and serum levels of the molecules evaluated in AS patients treated with anti-TNF. (**A**) Correlation between total hip BMD and DKK-1; (**B**) Correlation between total hip BMD and SOST; (**C**) Correlation between total hip BMD and BMP-6; (**D**) Correlation between total hip BMD and TNFα; (**E**) Correlation between total hip BMD and IL-17. * Statistical significance *p* ≤ 0.05.

In group 1 (bDMARDs anti-TNF + csDMARDs) spine BMD showed an association with serum DKK-1 (Rho = 0.369, *p* = 0.0225) and IL-17 (Rho = 0.438, *p* = 0.0108). Hip BMD is associated with serum DKK-1 (Rho = 0.346, *p* = 0.033), with serum BMP-6 (Rho = −0.736, *p* = 0.0128), and with IL-17 (Rho = 0.392, *p* = 0.024), Figure 3.

Additionally, a binary multiple logistic regression analysis was performed to explore factors associated with the low BMD in AS patients. In the intro model, the dependent variable was low BMD (yes/no), adjusting for potential confounders, including age, BMI, glucocorticoid dose, serum biomarker levels, and the use of anti-TNF bDMARD therapy. None of the evaluated variables were significantly associated with low BMD in AS patients, Supplementary Table S1.

## 4. Discussion

We identified that almost one of each three patients with AS and one of each five patients with AS had low BMD in the lumbar spine and total hips, respectively. These results are similar to those reported in a systematic review of seven studies that found a decrease in BMD that goes from 19% to 62% in AS [14]. These findings are relevant because low BMD is associated with osteoporotic fractures; the risk of vertebral fractures in these patients is almost two-fold that observed in controls [15].

When we assessed the BMD in two regions, we found that low BMD is mainly observed in the lumbar spine (31.6%) compared to the total hips (21.1%) of patients with AS. This finding has also been reported previously by other authors [16]. However, in our study, we observed differences in the BMD of total hips in patients with AS, compared with controls. Other authors found no differences in the risk of hip fractures [15]. This low BMD in AS may be due to multiple factors, including the persistence of chronic inflammation observed in AS, as well as the fact that one-third of these patients were using glucocorticoids, resulting in a deterioration of bone quality [17].

Regarding the anti-TNF therapies, we did not find statistical differences in the prevalence of low BMD in the lumbar spine or total hips of patients with AS treated with anti-TNF agents vs. those treated with csDMARDs. Moreover, we found that anti-TNF agents do not modify BMD; in spite of this, low BMD frequency is similar in the double therapy group, which is similar to what was reported in previous studies [18].

In a review of seven longitudinal studies on one clinical trial, the use of anti-TNF agents showed a mild increase in the BMD of the lumbar spine and the total hips [19]. Nevertheless, no comparisons were performed in that systematic review with patients treated with csDMARDs. Therefore, our study provides evidence that probably the effect of both therapies in remitting inflammation leads to an improvement of BMD.

In our institution, the use of anti-TNF is not employed as the first line of treatment in AS patients; these agents are used in patients who are refractory to csDMARDs; therefore, these patients had a more aggressive disease course [3].

Patients with AS treated with anti-TNF agents had lower levels of BMP-6 compared to controls, but no differences were observed in BMP-6 between AS treated with anti-TNF agents vs. csDMARD. This suggests that AS patients may exhibit increased bone fragility, independent of the treatment they receive. The bone morphogenetic protein (BMP) family is a large group of glycoproteins that play a crucial role in bone development. BMPs are important signaling molecules within the TGF-β family [20]. Among the various subtypes, BMP-6 is particularly notable for its bone formation-promoting effects, as it stimulates osteoblast activity. Elevated levels of circulating BMP-6 have been observed in patients with AS, as reducing TNF-α activity is associated with an increase in bone morphogenetic proteins through regulation of the non-canonical Wnt/β-catenin signaling pathway [21]. The cessation of TNF-α activity indirectly modulates BMP-6 levels, as TNF-α inhibits the transcriptional factor Smad, which alters BMP’s canonical pathway, which explains BMP-6 behavior in patients with anti-TNF therapy [22]. Also, long-term treatment with anti-TNF agents has been shown to promote the balance of bone turnover in AS [23,24,25]. TNF-α levels were higher in patients with AS and anti-TNF therapy than in subjects without AS, which is different from what has been reported on rheumatic diseases with anti-TNF therapy. This can be explained by the capacity of Etanercept to bind the soluble trimeric form of TNF-α at a hotspot located at the junction of each subunit [26], inhibiting its interaction with membrane receptors, thereby increasing its levels in circulation [27]. In addition to this, it has been reported that soluble TNF-α is increased in ankylosing spondylitis patients > 2 months with Etanercept therapy and for rheumatoid arthritis > 2 months [28]. It has been demonstrated that Adalimumab cannot restore intracellular TNF-α to normal values after a long period of therapy, whereas Infliximab has been shown to regulate TNFα levels, similar to our study [29].

No significant differences were observed in the levels of the antagonistic Wnt/β-catenin pathway proteins (DKK-1 and SOST) between patients receiving anti-TNF agents plus csDMARDs and those receiving only csDMARDs, consistent with findings reported by Ustun et al., suggesting that anti-TNF agents had no significant effect on the serum levels of these molecules [30]. Previous studies have reported that serum DKK-1 and SOST are not affected by treatment with anti-TNF agents in spondylopathies and are only modified depending on age [31], while other studies report that in patients with AS treated with TNF inhibitors, serum levels of DKK-1 are increased [32]; the results remain controversial.

IL-17 levels showed no statistical difference between groups, which concurs with reports on studies in other autoinflammatory conditions [33,34], suggesting that anti-TNF agents play no major role on IL-17 activity.

By performing bivariate correlations with protein levels and BMD, we found a positive correlation of DKK-1 serum levels with lumbar spine BMD, which is consistent with previous reports that elevated DKK-1 levels reduce osteogenic differentiation, whereas low levels promote activation of the Wnt/β-catenin signaling pathway [35]. However, a negative correlation with the same anatomical region has been reported in other populations with autoimmune diseases without anti-TNF treatment [36]; then, the use of biological treatments could influence the behavior of DKK-1.

The results in our study show a positive correlation between IL-17 levels and bone mineral density in both anatomical regions, contrary to the reported antagonistic effect of IL-17 on bone mineral density [37]. This has been supported in preclinical studies, since IL-17 can decrease adipogenesis, which translates into an increase in bone mineral density [38] in addition to being a cytokine that plays a role that depends on the conditions in which the organism is found, since it can participate in the signaling of bone formation [39].

A negative correlation was observed between serum BMP-6 levels and total hip BMD, which may be due to the continuous activation of the canonical BMP-6 pathway, potentially leading to a loss of osteoblast function [40].

Also, we observed a negative correlation between BMP-6 and SOST serum levels; this instance can be explained by a modulation by the non-canonical Wnt signaling pathway [41].

### Limitations and Strengths of the Study

This study has limitations that should be considered in future studies. The cross-sectional design does not allow determining whether the observed alterations in serum levels of DKK-1, SOST, BMP-6, and bone mineral density preceded or resulted from disease activity, nor does it allow assessing potential changes over time or the modulatory effects of anti-TNF therapy, alone or in combination with csDMARDs, in patients with AS. In addition, the absence of direct data on bone fractures represents a limitation, as such measurements do not always reflect actual fracture risk. Consequently, causal conclusions about the impact of these treatments on bone health cannot be drawn. Furthermore, the study sample might not extrapolate to the general AS population due to clinical heterogeneity among participants, limiting the generalizability of the findings. Because of this, the need for longitudinal studies monitoring biomarker dynamics and BMD changes over time in patients with AS is highlighted, allowing for a more precise assessment of therapeutic effects and their temporal relationship.

Despite these limitations, this study has important strengths. The comparative analysis of patients treated with anti-TNF bDMARDs versus csDMARDs provides valuable information on the differential effects of these therapies on bone turnover and bone mineral density (BMD) in AS. To our knowledge, this is one of the first studies to integrate bone turnover biomarker measurements, BMD assessment, and drug treatment analysis in patients with AS. The use of standardized biomarker assays and densitometric techniques further improves the robustness and reliability of the findings. Altogether, these results underscore the value of bone densitometry in diagnosis and bone quality monitoring, while highlighting the need for further studies evaluating the impact of novel biologic therapies on bone metabolism in AS.

## 5. Conclusions

Low BMD (osteopenia or osteoporosis) in the lumbar spine was observed in one-third of patients with AS (31.6%), and one-fifth of these patients also had low BMD in the hips (21.1%). AS patients had lower levels of BMP-6 compared to healthy donors and almost 2-fold serum levels of TNF-α. In this study, the combination of anti-TNF bDMARD therapy and csDMARDs was not significantly associated with serum levels of DKK-1, SOST, BMP-6, and BMD compared to those treated with csDMARD monotherapy in patients with AS. Interestingly, although mean BMD values did not differ between groups, a higher proportion of patients with AS had a low BMD compared with controls. Disease activity and functional loss seem to be equal in both groups, which supports the fact that bDMARDs anti-TNF are used to delay the wear and tear produced by chronic inflammation in AS. However, the absence of observed differences does not rule out possible dynamic changes in bone-related biomarkers depending on treatment duration or patient characteristics, and therefore future longitudinal studies are proposed.

## Figures and Tables

**Table 1:** Characteristics of AS patients.

Characteristics	AS Patients (*n* = 76)
*Sociodemographic Characteristics*	
Male Gender, *n* (%)	46 (60.5)
Age (Years), Median (Min–Max)	49 (18–69)
BMI (kg/m^2^), Median (Min–Max)	26.5 (16.40–40.86)
*Disease Characteristics*	
Disease Evolution (Years), Median (Min–Max)	10 (1–33)
BASDAI (Score), Median (Min–Max)	5.35 (0.4–9.5)
BASFI (Score), Median (Min–Max)	3.9 (0–10)
*Bone Mineral Density*	
BMD in Lumbar Spine, L1–L4 (gr/cm^2^), Median (Min–Max)	1.11 (0.75–1.68)
Low BMD in Lumbar Spine, L1–L4, *n* (%)	24 (31.6)
BMD in Total Hip (gr/cm^2^), Median (Min–Max)	0.94 (0.63–1.30)
Low BMD in Total Hip, *n* (%)	16 (21.1)
Results of BMD, *n* (%)	
Osteoporosis, *n* (%)	8 (10.5)
Osteopenia, *n* (%)	16 (21.1)

Quantitative values are expressed as medians and min-max, while qualitative values are expressed as frequencies and percentages. AS: Ankylosing Spondylitis: BMI: Body Mass Index (kg/m^2^); BASDAI: Bath Ankylosing Spondylitis Disease Activity Index; BASFI: Bath Ankylosing Spondylitis Functional Index; BMD: Bone Mineral Density. Low BMD includes osteoporosis and osteopenia.

**Table 2:** Comparison of clinical characteristics in patients with AS vs. healthy donors.

Characteristics	AS Patients (*n* = 76)	Healthy Donors (*n* = 30)	*p*
*Sociodemographic Characteristics*			
Male Gender, *n* (%)	46 (60.5)	16 (53.3)	0.498
Age (Years), Median (Min–Max)	49 (18–69)	46 (23–64)	0.096
BMI (kg/m^2^), Median (Min–Max)	26.5 (16.40–40.86)	28.75 (20.19–40.96)	0.014
*Bone Mineral Density*			
BMD in Lumbar Spine, L1–L4 (gr/cm^2^), Median (Min–Max)	1.11 (0.75–1.68)	1.19 (0.92–1.44)	0.101
Low BMD in Lumbar Spine, L1–L4, *n* (%)	24 (31.6)	2 (6.7)	0.024
BMD in Total Hips (gr/cm^2^), Median (Min–Max)	0.94 (0.634–1.30)	1.06 (0.81–1.34)	<0.001
Low BMD in Total Hips, *n* (%)	16 (21.1)	1 (3.3)	0.077
*Serum Bone turnover markers and cytokines levels*			
DKK-1, (pg/mL), Median (Min–Max)	815.90 (13.77–8434.50)	862.60 (368.0–1710.0)	0.742
SOST, (pg/mL), Median (Min–Max)	65.82 (6.48–357.32)	67.85 (17.54–167.80)	0.757
BMP-6, (pg/mL), Median (Min–Max)	2.05 (0.41–52.77)	21.44 (12.17–50.0)	0.001
TNF-α, (pg/mL), Median (Min–Max)	8.87 (0.01–938.19)	4.66 (0.01–18.4)	<0.001
IL-17, (pg/mL), Median (Min–Max)	5.44 (1.65–69.07)	4.92 (2.40–28.19)	0.371

Quantitative values are expressed as medians and min-max, while qualitative values are expressed as frequencies and percentages. Comparatives were assessed between Ankylosing Spondylitis (AS) vs. healthy donors using the Mann–Whitney U test. Comparatives between qualitative variables were assessed using the chi-square. BMI: Body Mass Index (kg/m^2^); BMD: Bone Mineral Density. Low BMD includes osteoporosis and osteopenia. DKK-1: Dickoppf 1. SOST: Sclerostin. BMP-6: Bone Morphogenetic Protein 6. TNF-α: Tumor Necrosis Factor-Alpha. IL-17: Interleukin 17.

**Table 3:** Comparison of serum DKK-1, SOST, BMP-6, and cytokines in AS study groups.

Characteristics	Anti-TNF + csDMARD (*n* = 38)	csDMARD (*n* = 38)	*p*
*Sociodemographic Characteristics*			
Male Gender, *n* (%)	27 (71.1)	19 (50)	0.050
Age (years), Median (Min–Max)	43 (18–69)	53 (24–66)	0.007
BMI (kg/m^2^), Median (Min–Max)	25.88 (16.59–40.86)	27.38 (16.40–39.94)	0.309
Disease Evolution (Years), Median (Min–Max)	10 (1–33)	10 (1–30)	0.625
*Clinical Disease Index*			
BASDAI (Score), Median (Min–Max)	5.1 (0.4–9.5)	5.9 (1.4–9.2)	0.285
BASFI (Score), Median (Min–Max)	3.7 (0–9.5)	3.9 (0.8–10.0)	0.394
*Bone Mineral Density*			
BMD in Lumbar Spine, L1–L4 (gr/cm^2^), Median (Min–Max)	1.11 (0.75–1.68)	1.10 (0.79–1.55)	0.930
Low BMD in Lumbar Spine, L1–L4	12 (31.6)	12 (31.6)	1.000
BMD in Total Hips (gr/cm^2^), Median (Min–Max)	0.94 (0.66–1.30)	0.93 (0.63–1.26)	0.876
Low BMD in Total Hips, *n* (%)	8 (21.1)	8 (21.1)	1.000
Low BMD in Any Region, *n* (%)	14 (36.8)	14 (36.8)	1.000
*Serum Bone Turnover Markers and Cytokine Levels*			
DKK-1, (pg/mL), Median (Min–Max)	868.45 (13.77–6748.21)	730.85 (31.30–8434.54)	0.815
SOST, (pg/mL), Median (Min–Max)	63.09 (7.19–299.12)	65.82 (6.48–357.32)	0.771
BMP-6, (pg/mL), Median (Min–Max)	3.34 (0.41–52.77)	1.60 (0.58–41.16)	0.451
TNF-α, (pg/mL), Median (Min–Max)	17.42 (0.013–350.60)	7.02 (0.72–938.19)	0.017

Quantitative values are expressed as medians and min-max, while qualitative values are expressed as frequencies and percentages. Comparatives were assessed between group 1 (bDMARDs anti-TNF + csDMARDs) using Mann–Whitney U test. Comparatives between qualitative variables were assessed using the chi-square. BMI: Body Mass Index. BASDAI: Bath Ankylosing Spondylitis Disease Activity Index. BASFI: Bath Ankylosing Spondylitis Functional Index. BMD: Bone Mineral Density. Low BMD includes osteoporosis and osteopenia. DKK-1: Dickoppf 1. SOST: Sclerostin. BMP-6: Bone Morphogenetic Protein 6. TNF-α: Tumor Necrosis Factor-Alpha. IL-17: Interleukin 17.

## Data Availability

The datasets produced in this study can be accessed by contacting the corresponding authors.

## Supplementary Materials

The following supporting information can be downloaded at: https://www.mdpi.com/article/10.3390/medsci13030189/s1, Table S1: Factors associated with low BMD in AS patients in the binary multiple logistic regression.

## Abbreviations

The following abbreviations are used in this manuscript:

AS	ankylosing spondylitis
csDMARDs	conventional synthetic disease-modifying antirheumatic drugs
bDMARDs	biologic disease-modifying antirheumatic drugs
TNF-α	tumor necrosis factor-alpha
IL-17	interleukin-17
BMD	bone mineral density
DKK-1	dickkopf-1
SOST	sclerostin
BMP-6	bone morphogenetic protein-6
INTEC	Institute of Experimental and Clinical Therapeutics
BASDAI	bath ankylosing spondylitis disease activity index
BASFI	bath ankylosing spondylitis functional index
DXA	dual-energy X-ray absorptiometry
ELISA	enzyme-linked immunosorbent assay
NSAID	non-steroidal anti-inflammatory drugs
BMI	body mass index
