# Supplementary material for: Bone Mineral Density and Serum Levels of Bone Remodeling Markers in Ankylosing Spondylitis Treated with Anti TNF-α Agents
Source: Med Sci (Basel). 2025 Sep 13;13(3):189. doi: 10.3390/medsci13030189 (PMC12452767; doi:10.3390/medsci13030189)
Supplement: Supplementary file 1 [file medsci-13-00189-s001.zip › medsci-3838316-supplementary.pdf]

Supplementary Table S1. Factors associated with low BMD in AS patients in the binary multiple logistic regression.

Variables	OR	IC95%	*p*
Age, (years)	1.077	0.890 – 1.303	0.448
Glucocorticoid dose, (mg/day)	0.689	0.269 – 1.770	0.439
DKK-1 serum levels, (pg/mL)	1.000	0.999 – 1.001	0.639
SOST serum levels, (pg/mL)	0.970	0.918 – 1.025	0.276
BMP-6 serum levels, (pg/mL)	0.986	0.869 – 1.119	0.826
IL-17 serum levels, (pg/mL)	1.026	0.758 – 1.389	0.867
TNFα serum levels, (pg/mL)	0.998	0.985 -1.012	0.817
Anti TNF bDMARD treatment, (yes/no)	3.735	0.100 – 139.606	0.476
BMI, (kg/m^2)	Not included in the model		

Multivariate analysis: Binary Multiple Logistic Regression analysis. *Dependent variable:* low BMD (osteopenia or osteoporosis). This model was adjusted by age, glucocorticoid dose, DKK-1, SOST, BMP-6, IL-17, TNFα, and Anti TNF bDMARD treatment. Covariates included in this analysis were those variables with statistical significance in the univariate analysis or were considered with biological plausibility. BMD: Bone Mineral Density; DKK-1: Dickoppf 1; SOST: Sclerostin; BMP-6: Bone Morphogenetic Protein 6; IL-17: Inter-leukin 17; TNF-α: Tumor Necrosis Factor-Alpha. BMI: Body Mass Index.
